# Bis(2-naphthyl­meth­yl)diphenyl­silane

**DOI:** 10.1107/S1600536809047412

**Published:** 2009-12-12

**Authors:** Thomas Blake Monroe, Andy A. Thomas, Daniel S. Jones, Craig A. Ogle

**Affiliations:** a Department of Chemistry, The University of North Carolina at Charlotte, 9201 University City Blvd, Charlotte, NC 28223, USA

## Abstract

The title compound, C_34_H_28_Si, was prepared as an inter­nal standard for diffusion-ordered NMR spectroscopy. The four ligands are arranged tetra­hedrally around the Si atom. The two naphthalene systems are nearly perpendicular, making an angle of 86.42 (4)° with one another. A naphthalene system and a phenyl ring are also nearly perpendicular, making an angle of 86.18 (6)° with one another. In the crystal, the mol­ecules pack in columns parallel to the *a* axis, and exhibit arene C—H⋯π(arene) inter­actions both within and between columns.

## Related literature

For applications of the title compound related to NMR spectroscopy, see: Li *et al.* (2009 ▶). A search of the Cambridge Structural Database (Allen, 2002 ▶; CONQUEST; Bruno *et al.*, 2002 ▶) yielded no comparable structures.
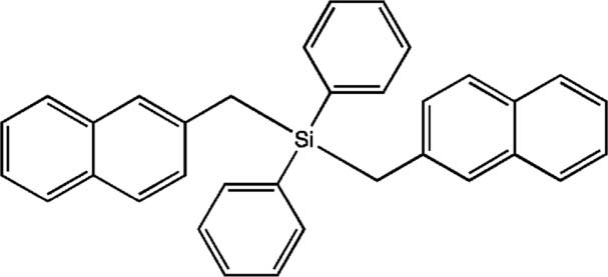


## Experimental

### 

#### Crystal data

C_34_H_28_Si
*M* *_r_* = 464.65
Triclinic, 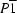

*a* = 9.4884 (14) Å
*b* = 11.0673 (13) Å
*c* = 13.3450 (15) Å
α = 75.820 (9)°
β = 83.767 (11)°
γ = 70.575 (11)°
*V* = 1280.8 (3) Å^3^
*Z* = 2
Cu *K*α radiation
μ = 0.94 mm^−1^
*T* = 295 K
0.36 × 0.20 × 0.05 mm

#### Data collection

Enraf–Nonius CAD-4 diffractometer
Absorption correction: analytical (Alcock, 1970 ▶) *T*_min_ = 0.814, *T*_max_ = 0.954
9101 measured reflections
4618 independent reflections
3060 reflections with *I* > 2σ(*I*)
*R*_int_ = 0.045
3 standard reflections every 165 reflections intensity decay: 1%

#### Refinement

*R*[*F* ^2^ > 2σ(*F* ^2^)] = 0.037
*wR*(*F* ^2^) = 0.102
*S* = 1.01
4618 reflections
333 parameters
H atoms treated by a mixture of independent and constrained refinement
Δρ_max_ = 0.17 e Å^−3^
Δρ_min_ = −0.20 e Å^−3^

### 

Data collection: *CAD-4 EXPRESS* (Enraf–Nonius, 1994 ▶); cell refinement: *CAD-4 EXPRESS*; data reduction: *XCAD4* (Harms & Wocadlo, 1995 ▶); program(s) used to solve structure: *SHELXS97* (Sheldrick, 2008 ▶); program(s) used to refine structure: *SHELXL97* (Sheldrick, 2008 ▶); molecular graphics: *ORTEP-3 for Windows* (Farrugia, 1997 ▶) and *Mercury* (Macrae *et al.*, 2006 ▶); software used to prepare material for publication: *WinGX* (Farrugia, 1999 ▶).

## Supplementary Material

Crystal structure: contains datablocks global, I. DOI: 10.1107/S1600536809047412/fl2263sup1.cif

Structure factors: contains datablocks I. DOI: 10.1107/S1600536809047412/fl2263Isup2.hkl

Additional supplementary materials:  crystallographic information; 3D view; checkCIF report

## Figures and Tables

**Table 1:** Arene C—H⋯π (arene) packing interactions (Å, °)

*D*—H⋯*A*	*D*—H	H⋯*A*	*D*⋯*A*	*D*—H⋯*A*
C3—H3⋯*Cg*1^i^	0.93	2.66	3.569 (2)	166
C5—H5⋯*Cg*2^ii^	0.93	2.88	3.664 (2)	143
C9—H9⋯*Cg*3^iii^	0.93	2.76	3.577 (2)	148

Symmetry codes: (i) 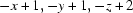; (ii) 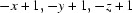; (iii) 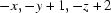. *Cg*1, *Cg*2, and *Cg*3 are the centroids of the C7–C12, C14–C17/C22/C23 and  C27–C32 rings, respectively.

**Fig. 1.**
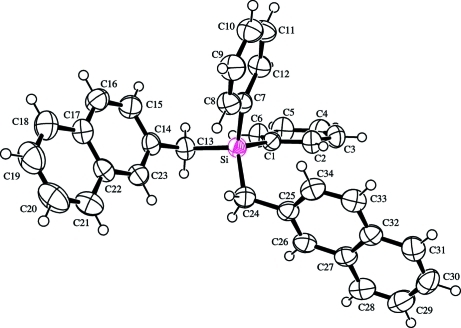
View of title compound (50% probability displacement ellipsoids)

**Fig. 2.**
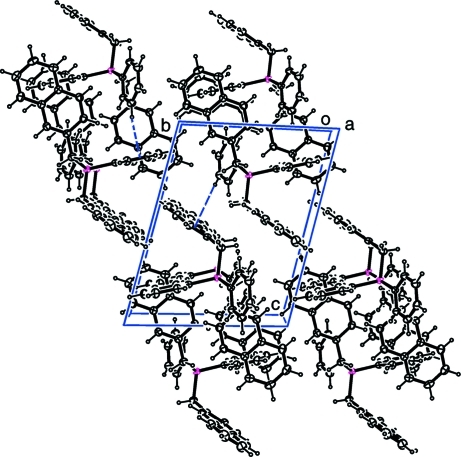
Diagram showing how molecules of the title compound form columns parallel to the a axis. Naphthyl-phenyl C—H···π interactions between columns and phenyl-phenyl C—H···π interactions within columns are illustrated

**Fig. 3.**
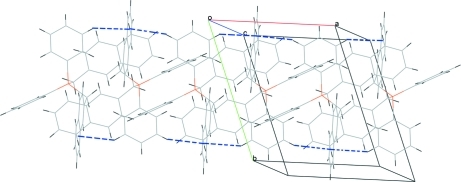
Diagram showing naphthyl-phenyl C—H···π interactions within columns

**Fig. 4.**
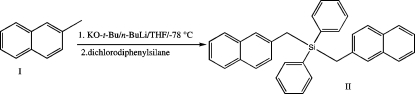
Synthesis scheme

## supplementary crystallographic information

### Comment

The title compound was prepared as an internal standard for diffusion-ordered NMR spectroscopy. A recent paper on this subject (Li *et al.*, 2009) suggests an internal standard method for correlating diffusion coefficients with formula weights. The title compound was chosen because its shape both approximates that of a spheroid and is similar to that of the species being studied. In addition, it neither reacts with the species under study nor gives interfering NMR signals.

The ligands are arranged tetrahedrally around the silicon atom. The two naphthalene rings of the title compound are nearly perpendicular, making an angle of 86.42 (4)° with one another. A naphthalene ring and a phenyl ring are also nearly perpendicular, making an angle of 86.18 (6)° with one another. The angle between the phenyl rings is 74.35 (7)°. The molecules pack in columns parallel to the *a* axis and exhibit arene C—H··· arene π interactions both within and between columns. These interactions between a phenyl of one molecule and a proximal aromatic ring of a naphthyl on a molecule in an adjacent column are 2.879 (3) Å in length (Figure 2). The interactions between two phenyls of two adjacent molecules in the same column are 2.659 (2) Å in length (Figure 2). The interactions between a phenyl of one molecule and a distal aromatic ring of a naphthyl on an adjacent molecule in the same column are 2.757 (2) Å in length (Figure 3).

A search of the Cambridge Structural Database [Version 5.30 (Allen, 2002); CONQUEST (Bruno *et al.*, 2002)] yielded no comparable structures. The search fragment used consisted of two naphthalene rings coordinated to Si, both with and without the methylene bridges.

### Experimental

A dry, 250 ml Schlenk flask, equipped with a magnetic stir bar, was charged with 2-methylnaphthalene (I) (4.26 g, 300 mmol) and potassium *tert*-butoxide (3.92 g, 350 mmol). The Schlenk flask was purged with nitrogen. Freshly distilled THF (100 ml) was added and the reaction was cooled to -78 °C. *n*-BuLi (15.2 ml, 2.3 *M*) was then added dropwise. The Schlenk flask was then capped and kept at -40 °C overnight. The solution was again cooled to -78 °C and 2.09 ml (2.52 g, 100 mmol) of dichlorodiphenylsilane was added dropwise. The reaction mixture was allowed to warm to room temperature and stirred for 2 h. The mixture was then quenched with deionized water and extracted three times with hexanes. The combined organic layers were dried with magnesium sulfate, filtered, and evaporated. Bulb-to-bulb distillation gave a tan solid, which was recrystallized from hexanes to yield colorless crystals of the title compound (II) (2.56 g, 55% recovery).

mp 98 - 100 °C; ^1^H NMR (DMSO-*d*_6_, 300 MHz): 2.86(*s*), 6.97 (*d*), 7.32 (*m*), 7.41(*m*), 7.58 (*m*), 7.75 (*d*) p.p.m.. ^13^C NMR (DMSO-*d*_6_, 300 MHz): 21.92, 125.83, 126.27, 126.73, 127.10, 127.36, 127.71, 128.19, 129.59, 130.65, 133.06, 134.18, 135.14, 136.11 p.p.m.. GC/MS (70ev) m/*z*: 464.3, 323.2, 245.1, 215.1,193.1, 167.1, 141.1, 105.0.

### Refinement

The four H atoms bonded to the methylene carbons were located in a difference map and refined. All other H atoms were constrained using a riding model; the C—H bond lengths were fixed at 0.93 Å with *U*_iso_(H) = 1.2 *U*_eq_(C).

### Crystal data

**Table:**

C_34_H_28_Si	*Z* = 2
*M**_r_* = 464.65	*F*(000) = 492
Triclinic, *P*1	*D*_x_ = 1.205 Mg m^−^^3^
Hall symbol: -P 1	Cu *K*α radiation, λ = 1.54184 Å
*a* = 9.4884 (14) Å	Cell parameters from 25 reflections
*b* = 11.0673 (13) Å	θ = 9.7–40.3°
*c* = 13.3450 (15) Å	µ = 0.94 mm^−^^1^
α = 75.820 (9)°	*T* = 295 K
β = 83.767 (11)°	Prism, colorless
γ = 70.575 (11)°	0.36 × 0.20 × 0.05 mm
*V* = 1280.8 (3) Å^3^	

### Data collection

**Table:**

Enraf–Nonius CAD-4 diffractometer	*R*_int_ = 0.045
Non–profiled ω/2θ scans	θ_max_ = 67.4°, θ_min_ = 3.4°
Absorption correction: analytical (see. N.W. Alcock (1970))	*h* = −11→11
*T*_min_ = 0.814, *T*_max_ = 0.954	*k* = −13→13
9101 measured reflections	*l* = −15→15
4618 independent reflections	3 standard reflections every 165 reflections
3060 reflections with *I* > 2σ(*I*)	intensity decay: 1%

### Refinement

**Table:**

Refinement on *F*^2^	Secondary atom site location: difference Fourier map
Least-squares matrix: full	Hydrogen site location: inferred from neighbouring sites
*R*[*F*^2^ > 2σ(*F*^2^)] = 0.037	H atoms treated by a mixture of independent and constrained refinement
*wR*(*F*^2^) = 0.102	*w* = 1/[σ^2^(*F*_o_^2^) + (0.0422*P*)^2^ + 0.1595*P*] where *P* = (*F*_o_^2^ + 2*F*_c_^2^)/3
*S* = 1.00	(Δ/σ)_max_ = 0.001
4618 reflections	Δρ_max_ = 0.17 e Å^−^^3^
333 parameters	Δρ_min_ = −0.20 e Å^−^^3^
0 restraints	Extinction correction: *SHELXL97* (Sheldrick, 2008), Fc^*^=kFc[1+0.001xFc^2^λ^3^/sin(2θ)]^-1/4^
Primary atom site location: structure-invariant direct methods	Extinction coefficient: 0.0037 (4)

### Special details

**Table:**

Geometry. All e.s.d.'s (except the e.s.d. in the dihedral angle between two l.s. planes) are estimated using the full covariance matrix. The cell e.s.d.'s are taken into account individually in the estimation of e.s.d.'s in distances, angles and torsion angles; correlations between e.s.d.'s in cell parameters are only used when they are defined by crystal symmetry. An approximate (isotropic) treatment of cell e.s.d.'s is used for estimating e.s.d.'s involving l.s. planes.

### Fractional atomic coordinates and isotropic or equivalent isotropic displacement parameters (Å^2^)

**Table:**

	*x*	*y*	*z*	*U*_iso_*/*U*_eq_	
H24B	0.231 (2)	0.353 (2)	0.7647 (17)	0.069 (7)*	
H13A	0.348 (2)	0.458 (2)	0.5907 (16)	0.067 (7)*	
H24A	0.117 (3)	0.479 (2)	0.7954 (16)	0.063 (6)*	
H13B	0.422 (3)	0.572 (2)	0.5734 (18)	0.085 (8)*	
Si	0.34325 (6)	0.51722 (5)	0.74770 (4)	0.04382 (15)	
C1	0.5414 (2)	0.42670 (17)	0.78451 (14)	0.0431 (4)	
C7	0.2828 (2)	0.67956 (18)	0.78528 (13)	0.0429 (4)	
C14	0.1897 (2)	0.64223 (19)	0.55711 (14)	0.0484 (5)	
C25	0.2299 (2)	0.34952 (18)	0.91925 (15)	0.0468 (5)	
C12	0.3864 (2)	0.73768 (19)	0.80112 (15)	0.0507 (5)	
H12	0.4880	0.6936	0.7945	0.061*	
C22	−0.0752 (2)	0.7066 (2)	0.52307 (14)	0.0500 (5)	
C27	0.2921 (2)	0.15022 (19)	1.05667 (16)	0.0489 (5)	
C32	0.2569 (2)	0.22750 (19)	1.13133 (15)	0.0481 (5)	
C34	0.1945 (2)	0.42439 (19)	0.99582 (16)	0.0554 (5)	
H34	0.1610	0.5156	0.9760	0.067*	
C26	0.2781 (2)	0.21659 (19)	0.94994 (16)	0.0520 (5)	
H26	0.3030	0.1668	0.8999	0.062*	
C23	0.0584 (2)	0.6132 (2)	0.56567 (15)	0.0511 (5)	
H23	0.0574	0.5296	0.6005	0.061*	
C24	0.2150 (3)	0.4184 (2)	0.80615 (17)	0.0561 (5)	
C6	0.6578 (2)	0.3999 (2)	0.71170 (16)	0.0559 (5)	
H6	0.6359	0.4249	0.6419	0.067*	
C33	0.2086 (2)	0.3654 (2)	1.09779 (16)	0.0556 (5)	
H33	0.1859	0.4171	1.1465	0.067*	
C8	0.1324 (2)	0.75053 (19)	0.79637 (16)	0.0533 (5)	
H8	0.0602	0.7154	0.7860	0.064*	
C2	0.5820 (2)	0.38502 (19)	0.88809 (15)	0.0534 (5)	
H2	0.5081	0.4005	0.9395	0.064*	
C17	−0.0733 (2)	0.8326 (2)	0.46839 (15)	0.0538 (5)	
C11	0.3415 (2)	0.8586 (2)	0.82632 (17)	0.0610 (6)	
H11	0.4128	0.8951	0.8359	0.073*	
C13	0.3323 (2)	0.5450 (2)	0.60306 (15)	0.0533 (5)	
C5	0.8042 (2)	0.3374 (2)	0.73991 (18)	0.0667 (6)	
H5	0.8793	0.3220	0.6892	0.080*	
C9	0.0876 (2)	0.8709 (2)	0.82220 (17)	0.0610 (6)	
H9	−0.0138	0.9155	0.8294	0.073*	
C31	0.2693 (2)	0.1627 (2)	1.23757 (16)	0.0594 (5)	
H31	0.2464	0.2119	1.2880	0.071*	
C10	0.1923 (2)	0.9257 (2)	0.83749 (17)	0.0608 (6)	
H10	0.1623	1.0070	0.8551	0.073*	
C3	0.7281 (2)	0.3218 (2)	0.91611 (17)	0.0593 (5)	
H3	0.7513	0.2951	0.9857	0.071*	
C4	0.8400 (2)	0.2979 (2)	0.84222 (18)	0.0630 (6)	
H4	0.9389	0.2554	0.8613	0.076*	
C15	0.1884 (2)	0.7688 (2)	0.50217 (16)	0.0590 (5)	
H15	0.2761	0.7905	0.4955	0.071*	
C21	−0.2121 (3)	0.6793 (2)	0.5334 (2)	0.0698 (6)	
H21	−0.2162	0.5970	0.5690	0.084*	
C16	0.0625 (3)	0.8597 (2)	0.45870 (17)	0.0627 (6)	
H16	0.0662	0.9416	0.4217	0.075*	
C28	0.3387 (2)	0.0136 (2)	1.08931 (19)	0.0642 (6)	
H28	0.3629	−0.0377	1.0403	0.077*	
C30	0.3144 (2)	0.0290 (2)	1.26605 (18)	0.0678 (7)	
H30	0.3221	−0.0127	1.3357	0.081*	
C29	0.3492 (3)	−0.0456 (2)	1.1907 (2)	0.0709 (7)	
H29	0.3799	−0.1368	1.2107	0.085*	
C18	−0.2061 (3)	0.9261 (2)	0.42547 (18)	0.0712 (6)	
H18	−0.2051	1.0090	0.3891	0.085*	
C20	−0.3378 (3)	0.7729 (3)	0.4914 (2)	0.0857 (8)	
H20	−0.4274	0.7541	0.4993	0.103*	
C19	−0.3347 (3)	0.8966 (3)	0.4365 (2)	0.0856 (8)	
H19	−0.4214	0.9590	0.4075	0.103*	

### Atomic displacement parameters (Å^2^)

**Table:**

	*U*^11^	*U*^22^	*U*^33^	*U*^12^	*U*^13^	*U*^23^
Si	0.0394 (3)	0.0496 (3)	0.0420 (3)	−0.0157 (2)	−0.0023 (2)	−0.0067 (2)
C1	0.0433 (11)	0.0416 (10)	0.0431 (10)	−0.0136 (8)	−0.0019 (8)	−0.0067 (8)
C7	0.0369 (10)	0.0512 (10)	0.0399 (10)	−0.0163 (8)	−0.0019 (8)	−0.0052 (8)
C14	0.0488 (12)	0.0609 (12)	0.0367 (10)	−0.0181 (10)	−0.0011 (8)	−0.0121 (8)
C25	0.0392 (11)	0.0510 (11)	0.0520 (11)	−0.0213 (9)	0.0014 (9)	−0.0061 (9)
C12	0.0329 (10)	0.0570 (12)	0.0618 (12)	−0.0129 (9)	0.0000 (9)	−0.0150 (9)
C22	0.0469 (12)	0.0643 (12)	0.0448 (11)	−0.0203 (10)	−0.0008 (9)	−0.0200 (9)
C27	0.0386 (11)	0.0518 (11)	0.0584 (12)	−0.0198 (9)	0.0030 (9)	−0.0104 (9)
C32	0.0334 (10)	0.0538 (11)	0.0573 (12)	−0.0175 (8)	0.0023 (8)	−0.0091 (9)
C34	0.0508 (13)	0.0489 (11)	0.0638 (13)	−0.0159 (9)	0.0040 (10)	−0.0099 (10)
C26	0.0490 (12)	0.0548 (12)	0.0590 (12)	−0.0244 (9)	0.0057 (9)	−0.0172 (10)
C23	0.0532 (12)	0.0548 (11)	0.0481 (11)	−0.0210 (10)	−0.0029 (9)	−0.0106 (9)
C24	0.0560 (14)	0.0611 (13)	0.0546 (13)	−0.0276 (12)	−0.0042 (10)	−0.0055 (10)
C6	0.0466 (12)	0.0663 (13)	0.0483 (11)	−0.0115 (10)	0.0003 (9)	−0.0110 (10)
C33	0.0512 (13)	0.0582 (12)	0.0603 (13)	−0.0190 (10)	0.0083 (10)	−0.0207 (10)
C8	0.0355 (11)	0.0580 (12)	0.0670 (13)	−0.0161 (9)	−0.0042 (9)	−0.0121 (10)
C2	0.0482 (12)	0.0630 (12)	0.0472 (11)	−0.0156 (10)	−0.0017 (9)	−0.0115 (9)
C17	0.0507 (12)	0.0613 (12)	0.0473 (11)	−0.0143 (10)	−0.0038 (9)	−0.0122 (9)
C11	0.0490 (13)	0.0616 (13)	0.0800 (15)	−0.0246 (11)	−0.0027 (11)	−0.0195 (11)
C13	0.0482 (13)	0.0628 (13)	0.0471 (11)	−0.0156 (10)	−0.0037 (9)	−0.0104 (10)
C5	0.0463 (13)	0.0776 (15)	0.0668 (15)	−0.0077 (11)	0.0054 (11)	−0.0188 (12)
C9	0.0388 (12)	0.0584 (13)	0.0793 (16)	−0.0065 (10)	−0.0044 (11)	−0.0151 (11)
C31	0.0437 (12)	0.0820 (15)	0.0564 (13)	−0.0271 (11)	0.0035 (10)	−0.0146 (11)
C10	0.0523 (13)	0.0514 (12)	0.0764 (15)	−0.0097 (10)	−0.0052 (11)	−0.0179 (11)
C3	0.0554 (13)	0.0637 (13)	0.0557 (12)	−0.0169 (11)	−0.0148 (10)	−0.0051 (10)
C4	0.0443 (12)	0.0586 (13)	0.0793 (16)	−0.0061 (10)	−0.0128 (11)	−0.0126 (11)
C15	0.0510 (13)	0.0677 (14)	0.0594 (13)	−0.0259 (11)	0.0018 (10)	−0.0078 (11)
C21	0.0592 (15)	0.0777 (16)	0.0843 (17)	−0.0321 (13)	−0.0011 (13)	−0.0252 (13)
C16	0.0600 (15)	0.0583 (13)	0.0653 (14)	−0.0217 (11)	−0.0031 (11)	−0.0012 (10)
C28	0.0576 (14)	0.0535 (12)	0.0812 (16)	−0.0199 (11)	0.0014 (12)	−0.0130 (11)
C30	0.0491 (13)	0.0839 (17)	0.0618 (14)	−0.0294 (12)	−0.0085 (11)	0.0142 (13)
C29	0.0606 (15)	0.0563 (13)	0.0880 (18)	−0.0216 (11)	−0.0068 (13)	0.0045 (13)
C18	0.0607 (16)	0.0724 (15)	0.0703 (15)	−0.0084 (12)	−0.0149 (12)	−0.0095 (12)
C20	0.0470 (15)	0.106 (2)	0.116 (2)	−0.0242 (14)	−0.0099 (14)	−0.0429 (18)
C19	0.0596 (17)	0.0880 (19)	0.104 (2)	−0.0052 (14)	−0.0254 (15)	−0.0265 (16)

### Geometric parameters (Å, °)

**Table:**

Si—C1	1.868 (2)	C8—H8	0.9300
Si—C7	1.8714 (19)	C2—C3	1.375 (3)
Si—C24	1.883 (2)	C2—H2	0.9300
Si—C13	1.887 (2)	C17—C16	1.402 (3)
C1—C6	1.393 (3)	C17—C18	1.409 (3)
C1—C2	1.399 (3)	C11—C10	1.373 (3)
C7—C8	1.393 (3)	C11—H11	0.9300
C7—C12	1.399 (3)	C13—H13A	0.98 (2)
C14—C23	1.373 (3)	C13—H13B	1.00 (3)
C14—C15	1.409 (3)	C5—C4	1.371 (3)
C14—C13	1.501 (3)	C5—H5	0.9300
C25—C26	1.356 (3)	C9—C10	1.379 (3)
C25—C34	1.414 (3)	C9—H9	0.9300
C25—C24	1.513 (3)	C31—C30	1.362 (3)
C12—C11	1.376 (3)	C31—H31	0.9300
C12—H12	0.9300	C10—H10	0.9300
C22—C17	1.412 (3)	C3—C4	1.372 (3)
C22—C23	1.413 (3)	C3—H3	0.9300
C22—C21	1.415 (3)	C4—H4	0.9300
C27—C28	1.395 (3)	C15—C16	1.357 (3)
C27—C32	1.411 (3)	C15—H15	0.9300
C27—C26	1.432 (3)	C21—C20	1.360 (3)
C32—C33	1.409 (3)	C21—H21	0.9300
C32—C31	1.421 (3)	C16—H16	0.9300
C34—C33	1.357 (3)	C28—C29	1.349 (3)
C34—H34	0.9300	C28—H28	0.9300
C26—H26	0.9300	C30—C29	1.398 (3)
C23—H23	0.9300	C30—H30	0.9300
C24—H24B	0.98 (2)	C29—H29	0.9300
C24—H24A	0.95 (2)	C18—C19	1.348 (3)
C6—C5	1.378 (3)	C18—H18	0.9300
C6—H6	0.9300	C20—C19	1.393 (4)
C33—H33	0.9300	C20—H20	0.9300
C8—C9	1.375 (3)	C19—H19	0.9300
			
C1—Si—C7	109.26 (8)	C16—C17—C18	122.4 (2)
C1—Si—C24	111.99 (10)	C16—C17—C22	118.1 (2)
C7—Si—C24	111.63 (10)	C18—C17—C22	119.5 (2)
C1—Si—C13	108.42 (9)	C10—C11—C12	120.6 (2)
C7—Si—C13	109.40 (9)	C10—C11—H11	119.7
C24—Si—C13	106.01 (11)	C12—C11—H11	119.7
C6—C1—C2	115.99 (18)	C14—C13—Si	115.41 (14)
C6—C1—Si	122.70 (15)	C14—C13—H13A	111.2 (13)
C2—C1—Si	121.28 (14)	Si—C13—H13A	104.7 (12)
C8—C7—C12	116.43 (17)	C14—C13—H13B	111.9 (14)
C8—C7—Si	121.88 (15)	Si—C13—H13B	105.3 (14)
C12—C7—Si	121.67 (14)	H13A—C13—H13B	107.8 (18)
C23—C14—C15	117.76 (19)	C4—C5—C6	120.4 (2)
C23—C14—C13	122.66 (19)	C4—C5—H5	119.8
C15—C14—C13	119.57 (19)	C6—C5—H5	119.8
C26—C25—C34	118.44 (18)	C8—C9—C10	120.23 (19)
C26—C25—C24	121.77 (19)	C8—C9—H9	119.9
C34—C25—C24	119.78 (18)	C10—C9—H9	119.9
C11—C12—C7	121.53 (18)	C30—C31—C32	120.4 (2)
C11—C12—H12	119.2	C30—C31—H31	119.8
C7—C12—H12	119.2	C32—C31—H31	119.8
C17—C22—C23	119.21 (19)	C11—C10—C9	119.1 (2)
C17—C22—C21	118.3 (2)	C11—C10—H10	120.4
C23—C22—C21	122.5 (2)	C9—C10—H10	120.4
C28—C27—C32	119.16 (19)	C4—C3—C2	120.5 (2)
C28—C27—C26	122.86 (19)	C4—C3—H3	119.7
C32—C27—C26	117.98 (17)	C2—C3—H3	119.7
C33—C32—C27	118.82 (18)	C5—C4—C3	119.1 (2)
C33—C32—C31	122.68 (19)	C5—C4—H4	120.4
C27—C32—C31	118.49 (18)	C3—C4—H4	120.4
C33—C34—C25	121.15 (18)	C16—C15—C14	121.7 (2)
C33—C34—H34	119.4	C16—C15—H15	119.2
C25—C34—H34	119.4	C14—C15—H15	119.2
C25—C26—C27	122.31 (18)	C20—C21—C22	120.2 (2)
C25—C26—H26	118.8	C20—C21—H21	119.9
C27—C26—H26	118.8	C22—C21—H21	119.9
C14—C23—C22	121.84 (19)	C15—C16—C17	121.3 (2)
C14—C23—H23	119.1	C15—C16—H16	119.3
C22—C23—H23	119.1	C17—C16—H16	119.3
C25—C24—Si	118.49 (15)	C29—C28—C27	121.2 (2)
C25—C24—H24B	108.9 (13)	C29—C28—H28	119.4
Si—C24—H24B	106.1 (13)	C27—C28—H28	119.4
C25—C24—H24A	109.2 (13)	C31—C30—C29	120.1 (2)
Si—C24—H24A	104.8 (13)	C31—C30—H30	119.9
H24B—C24—H24A	109.1 (18)	C29—C30—H30	119.9
C5—C6—C1	122.0 (2)	C28—C29—C30	120.6 (2)
C5—C6—H6	119.0	C28—C29—H29	119.7
C1—C6—H6	119.0	C30—C29—H29	119.7
C34—C33—C32	121.29 (19)	C19—C18—C17	120.7 (2)
C34—C33—H33	119.4	C19—C18—H18	119.6
C32—C33—H33	119.4	C17—C18—H18	119.6
C9—C8—C7	122.04 (19)	C21—C20—C19	121.1 (2)
C9—C8—H8	119.0	C21—C20—H20	119.4
C7—C8—H8	119.0	C19—C20—H20	119.4
C3—C2—C1	121.88 (19)	C18—C19—C20	120.2 (3)
C3—C2—H2	119.1	C18—C19—H19	119.9
C1—C2—H2	119.1	C20—C19—H19	119.9

### Hydrogen-bond geometry (Å, °)

**Table:**

*D*—H···*A*	*D*—H	H···*A*	*D*···*A*	*D*—H···*A*
C3—H3···Cg1^i^	0.93	2.66	3.569 (2)	166
C5—H5···Cg2^ii^	0.93	2.88	3.664 (2)	143
C9—H9···Cg3^iii^	0.93	2.76	3.577 (2)	148

Symmetry codes: (i) −*x*+1, −*y*+1, −*z*+2; (ii) −*x*+1, −*y*+1, −*z*+1; (iii) −*x*, −*y*+1, −*z*+2.
